# Risk factors for severe acute kidney injury among patients with rhabdomyolysis

**DOI:** 10.1186/s12882-020-02104-0

**Published:** 2020-11-23

**Authors:** Jia Yang, Jiaojiao Zhou, Xin Wang, Siwen Wang, Yi Tang, Lichuan Yang

**Affiliations:** 1 Division of Nephrology, Department of Medicine, West China Hospital of Sichuan University, Chengdu, 610041 Sichuan China; 2 Division of Ultrasound, West China Hospital of Sichuan University, Chengdu, Sichuan China; 3 Department of Pediatric Nephrology, West China Second University Hospital, Sichuan University, Chengdu, 610041 Sichuan China

**Keywords:** Acute kidney injury, Rhabdomyolysis, Risk factors

## Abstract

**Background:**

Acute kidney injury (AKI) is a life-threatening complication of rhabdomyolysis (RM). The aim of the present study was to assess patients at high risk for the occurrence of severe AKI defined as stage II or III of KDIGO classification and in-hospital mortality of AKI following RM.

**Methods:**

We performed a retrospective study of patients with creatine kinase levels > 1000 U/L, who were admitted to the West China Hospital of Sichuan University between January 2011 and March 2019. The sociodemographic, clinical and laboratory data of these patients were obtained from an electronic medical records database, and univariate and multivariate regression analyses were subsequently conducted.

**Results:**

For the 329 patients included in our study, the incidence of AKI was 61.4% and the proportion of stage I, stage II, stage III were 18.8, 14.9 and 66.3%, respectively. The overall mortality rate was 19.8%; furthermore, patients with AKI tended to have higher mortality rates than those without AKI (24.8% vs. 11.8%; *P* < 0.01). The clinical conditions most frequently associated with RM were trauma (28.3%), sepsis (14.6%), bee sting (12.8%), thoracic and abdominal surgery (11.2%) and exercise (7.0%). Furthermore, patients with RM resulting from sepsis, bee sting and acute alcoholism were more susceptible to severe AKI. The risk factors for the occurrence of stage II-III AKI among RM patients included hypertension (OR = 2.702), high levels of white blood cell count (OR = 1.054), increased triglycerides (OR = 1.260), low level of high-density lipoprotein cholesterol (OR = 0.318), elevated serum phosphorus (OR = 5.727), 5000<CK ≤ 10,000 U/L (OR = 2.617) and CK>10,000 U/L (OR = 8.093). Age ≥ 60 years (OR = 2.946), sepsis (OR = 3.206) and elevated prothrombin time (OR = 1.079) were independent risk factors for in-hospital mortality in RM patients with AKI.

**Conclusions:**

AKI is independently associated with mortality in patients with RM, and several risk factors were found to be associated with the occurrence of severe AKI and in-hospital mortality. These findings suggest that, to improve the quality of medical care, the early prevention of AKI should focus on high-risk patients and more effective management.

## Background

Rhabdomyolysis (RM) is a syndrome characterized by the disruption of skeletal muscle cell integrity, with subsequent release of intracellular components into the extracellular environment. There are numerous reported causes of RM, and the most frequently associated etiologies are trauma, immobilization, sepsis and surgery [1–3]. The clinical manifestations of RM (and their subsequent severity) vary based on the specific cause. These range from isolated elevation of laboratory indices, such as myoglobin and creatine kinase (CK), to life-threatening electrolyte disturbances and organ dysfunction [4, 5]. Acute kidney injury (AKI) is a common complication of RM, and the pathogenesis of RM-associated AKI includes tubular obstruction caused by myoglobin, myoglobin cytotoxicity by lipid peroxidation, and the production of reactive oxygen species. Intracellular components released into the circulation cause capillary damage, which leads to leakage and edema, secondary hypovolemia and decreased renal blood flow, and ultimately reduce renal function [2, 6]. The incidence of AKI is reported to be between 37.8 and 81.4% in patients with RM [7–11], and is independently associated with a 19.2–59.0% increase in mortality [9, 12, 13]. The occurrence of AKI is associated with a worse outcome in patients with RM, which increases medical burden; thus, the prevention and early diagnosis of AKI are critical to improving patient prognosis. Therefore, we conducted a retrospective analysis of 329 patients with RM to characterize the incidence of AKI in these patients, to identify independent risk factors of stage II-III AKI for prevention and early detection of it, and wanted to provide a useful indication of mortality risk in patients with AKI.

## Methods

### Study population

The present study was a retrospective analysis of data collected from the West China Hospital of Sichuan University, from patients admitted between January 2011 and March 2019. All patients over 18 years old with CK levels > 1000 U/L were eligible. The exclusion criteria were as follows: (i) Patients with pre-existing end-stage renal disease; (ii) patients who had received a kidney transplant; (iii) patients with abnormal CK elevation from acute myocardial infarction; and (iv) patients with incomplete data. To avoid bias among patients who were repeatedly admitted to hospital during the study period, only the first admission was included.

### Data collection

Patient sociodemographic, clinical and laboratory data were obtained from an electronic medical records database. Sociodemographic data included age, sex and date of admission. Clinical data included etiology, smoking history, alcoholism and chronic disease history (hypertension, diabetes and hyperlipidemia), the presence of sepsis and the outcomes (length of stay in hospital and mortality). Laboratory data comprised blood levels of CK, baseline biomarkers of renal function (serum creatinine (SCr), blood urea nitrogen (BUN), cystatin C and uric acid), levels of phosphate, calcium, potassium, aminotransferase, albumin, bilirubin, hemoglobin, lipoprotein and prothrombin time, as well as white blood cell (WBC) and platelet counts.

### Definition

In the present study, AKI was defined per the Kidney Disease Improving Global Outcomes criteria (KDIGO) [14]: An absolute increase in serum creatinine of ≥26.4 μmol/L within 48 h, or ≥ 50% baseline serum creatinine within 7 days. Due to incomplete urine output data, only the serum creatinine readings were available. For the baseline creatinine levels, we used the lowest value of serum creatinine recorded in the 2 days prior to admission, and if not available, the first serum creatinine reading within 2 days after admission [15]. In this study, the AKI was categorized as AKI I, II, III, respectively, according to increase in serum creatinine ≥26.4 μmol/L or increase to ≥1.5-fold to twofold from baseline, > twofold to threefold from baseline and > threefold from baseline or serum creatinine ≥354 μmol/L with an acute increase of at least 44 μmol/L. Individuals who receive renal replacement therapy were considered to have met the criteria of AKI III regardless of their serum creatinine value. The AKI stage was evaluated weekly, and the maximum degree was regarded as the final AKI stage. By reviewing the medical records of all patients, the etiologies of rhabdomyolysis and outcomes of patients during hospitalization were confirmed.

### Statistical analysis

Categorical covariates were recorded as frequency distributions and proportions. According to the distribution pattern, continuous variables were recorded as the mean ± standard deviation (SD). Univariate analysis was employed to predict disease etiology, using a binomial distribution; the Students t-test and Pearson χ2 test were used to estimate baseline characteristics. Independent predictors of AKI incidence and in-hospital mortality were evaluated by univariate and multivariate logistic regression, which were utilized to calculate odds ratios (ORs) and 95% confidence intervals. Only the significant risk factors identified by univariate analysis were considered for multiple regression analysis. The data were analyzed using SPSS 22.0 (IBM Corp., Armonk, NY, USA) and *P* < 0.05 (two-sided) was considered to indicate a statistically significant difference.

## Results

**Fig. 1:**
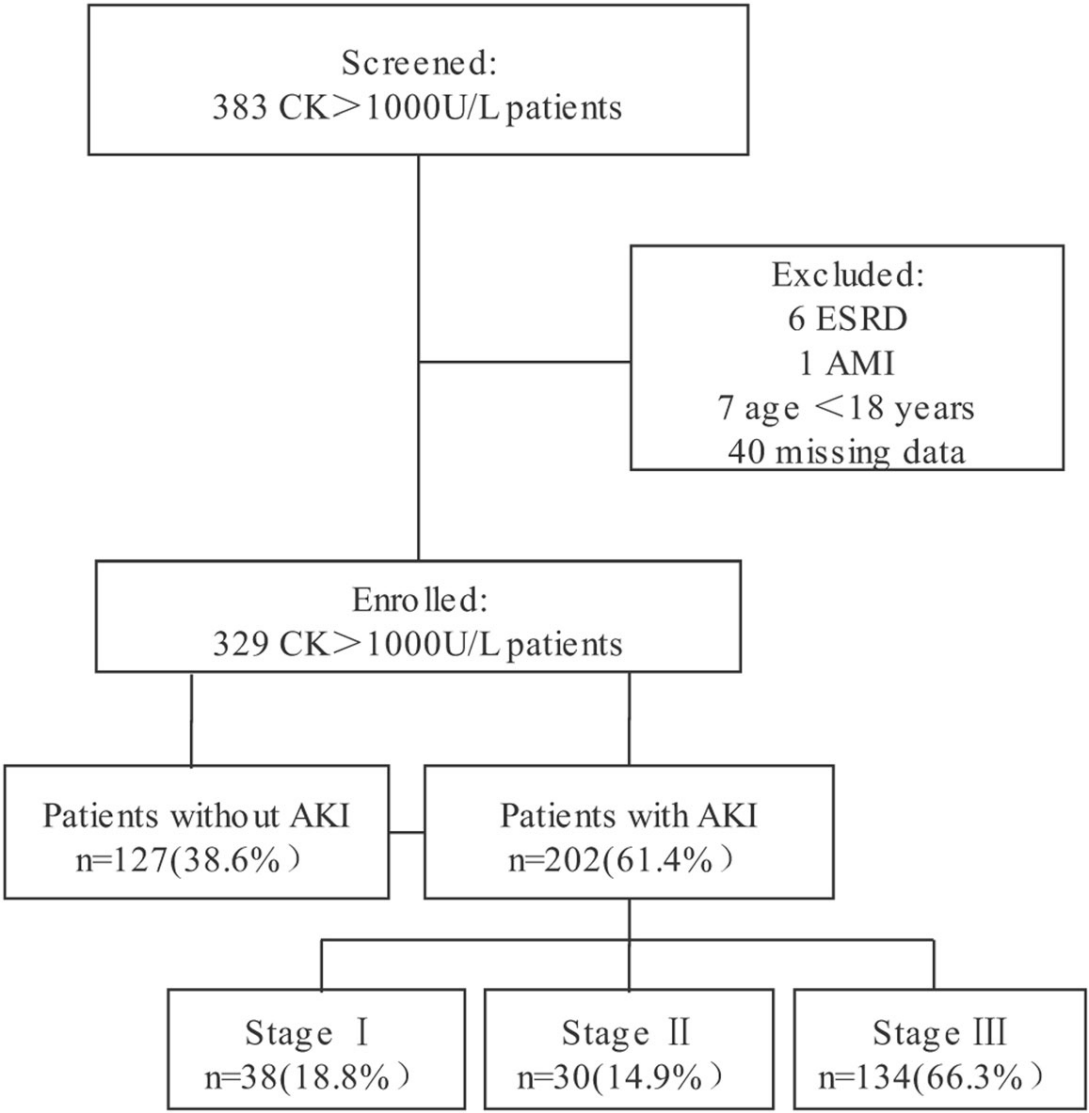
Study flow chart

In the present study, we identified 383 hospitalizations of adults with a CK level > 1000 U/L between January 2011 and March 2019. After excluding 6 patients with pre-existing end-stage renal disease or who had received a kidney transplant, 1 case of acute myocardial infraction, 7 patients aged< 18 years and 40 with incomplete data, the final study population included 329 patients (Fig. 1). There were 202 (61.4%) RM-induced AKI patients, among which 18.8% were classified as AKI stage I, 14.9% as AKI stage II and 66.3% as AKI stage III according to the KDIGO criteria. The patient age (mean ± SD) was 45.7 ± 15.9 years, and 74.8% of the patients were male. The overall in-hospital mortality rate was 19.8%, and patients with secondary AKI tended to exhibit higher mortality rates than those without AKI (24.8% vs. 11.8%, *P* < 0.01). The clinical conditions most frequently associated with RM were trauma (28.3%), sepsis (14.6%), bee sting (12.8%), thoracic and abdominal surgery (11.2%) and exercise (7.0%). Other causes are shown in Table 1. Patients with RM resulting from sepsis, bee sting and acute alcoholism were more susceptible to stage II-III AKI.

**Table 1:** The cause of 329 patients with rhabdomyolysis

Variables	Acute kidney injury stage II-III		***P***
	No, ***n*** = 165	Yes, ***n*** = 164	
Trauma, n (%)	67(72.0)	26(28.0)	0.000
Sepsis, n (%)	16(33.3)	32(66.7)	0.029
Bee sting, n (%)	3(7.1)	39(92.9)	0.000
Thoracic and abdominal operations, n (%)	25(67.6)	12(32.4)	0.047
Exercise, n (%)	11(47.8)	12(52.2)	1.000
Vascular surgery, n (%)	17(81.0)	4(19.0)	0.007
Acute alcoholism, n (%)	1(9.1)	10(90.9)	0.012
Drug, n (%)	3(50.0)	3(50.0)	1.000
Osteofascial compartment syndrome, n (%)	1(20.0)	4(80.0)	0.375
Myopathy, n (%)	3(75.0)	1(25.0)	0.625
Hyperthermia, n (%)	1(33.3)	2(66.7)	1.000
Orthopedic surgery, n (%)	2(66.7)	1(33.3)	1.000
Cardiac arrest, n (%)	2(100.0)	0(0.0)	1.000
Langoustine, n (%)	2(100.0)	0(0.0)	1.000
Seizure, n (%)	0(0.0)	1(100.0)	1.000
Diabetic ketoacidosis, n (%)	0(0.0)	1(100.0)	1.000
Unknown, n (%)	11(40.7)	16(59.3)	0.442

The baseline characteristics and their comparison between RM patients with or without stage II-III AKI are displayed in Table 2. Compared with the patients without stage II-III AKI, the proportions of patients aged ≥60 years, chronic alcoholism, hypertension, diabetes, sepsis, CK>10,000 U/L and those that succumbed to the disease were significantly higher in the stage II-III AKI group. Higher WBC counts, serum bilirubin, alanine aminotransferase (ALT), aspartate aminotransferase (AST), triglyceride, biomarkers of baseline renal function (SCr, BUN, cystatin C and uric acid), potassium, calcium and phosphorus levels were detected in patients with stage II-III AKI. The level of high-density lipoprotein cholesterol (HDLC) in patients with stage II-III AKI was lower than that in those without stage II-III AKI. However, in terms of gender, smoking history, hyperlipidemia, hemoglobin, blood platelet count, serum albumin, cholesterol, low-density lipoprotein cholesterol (LDLC), prothrombin time and length of stay, there were no significant differences between the two groups.

**Table 2:** Comparison of baseline characteristics between patients with or without stage II-III AKI

Variables	Acute kidney injury stage II-III		***p***
	No, ***n*** = 165	Yes, ***n*** = 164	
Age, mean ± SD, days	43.5 ± 16.0	47.8 ± 15.6	0.014
Age group, n (%)			0.024
Age ≤ 40	69 (41.8)	51 (31.1)	
40 < Age < 60	74 (44.8)	74 (45.1)	
Age ≥ 60	22 (13.3)	39 (23.8)	
Male gender, n (%)	129 (78.2)	117 (71.3)	0.153
Smoking, n (%)	60 (36.4)	59 (36.0)	0.942
Chronic alcoholism, n (%)	27 (16.4)	54 (32.9)	0.000
Comorbidity, n (%)			
Hypertension	15 (9.1)	36 (22.0)	0.001
Diabetes	7 (4.2)	20 (12.2)	0.009
Hyperlipidemia	12 (7.3)	17 (10.4)	0.322
Sepsis	83 (50.3)	112 (68.3)	0.001
Laboratory data, mean ± SD			
Hemoglobin (g/L)	105.5 ± 34.3	101.4 ± 39.7	0.314
Platelets (109/L)	147.4 ± 87.2	138.7 ± 84.8	0.358
WBC (109/L)	13.5 ± 7.4	17.3 ± 11.1	0.000
Total bilirubin (μmol/L)	27.8 ± 52.8	51.8 ± 84.4	0.002
Direct bilirubin (μmol/L)	14.7 ± 36.7	27.7 ± 47.4	0.006
ALT (U/L)	215.1 ± 336.2	453.6 ± 952.2	0.003
AST (U/L)	399.0 ± 565.5	1158.9 ± 2224.8	0.000
Albumin (g/L)	28.6 ± 9.2	29.6 ± 7.5	0.267
Baseline BUN (mmol/L)	6.9 ± 3.1	12.2 ± 7.7	0.000
Scr (μmol/L)	78.6 ± 24.7	185.5 ± 138.3	0.000
Cystatin C (μmol/L)	0.9 ± 0.4	2.2 ± 1.2	0.000
Uric acid (μmol/L)	252.2 ± 142.9	297.3 ± 148.0	0.005
Triglycerides (mmol/L)	1.5 ± 2.3	2.2 ± 2.6	0.016
Cholesterol (mmol/L)	3.1 ± 1.7	2.9 ± 1.8	0.487
HDLC (mmol/L)	0.9 ± 0.5	0.7 ± 0.4	0.000
LDLC (mmol/L)	1.6 ± 1.1	1.4 ± 1.1	0.098
Potassium (mmol/L)	3.9 ± 0.6	4.3 ± 0.9	0.000
Calcium (mmol/L)	2.0 ± 0.3	1.9 ± 0.3	0.022
Phosphate (mmol/L)	0.9 ± 0.4	1.6 ± 0.8	0.000
PT (s)	15.2 ± 4.2	16.1 ± 9.7	0.255
CK group, n (%)			0.000
1000 < CK ≤5000 (U/L)	109 (66.1)	40 (24.4)	
5000 < CK ≤10,000 (U/L)	26 (15.8)	28 (17.1)	
CK>10,000 (U/L)	30 (18.2)	96 (58.5)	
Outcome			
Length of stay, mean ± SD, days	29.4 ± 27.5	26.6 ± 22.1	0.304
Mortality, n (%)	23 (13.9)	42 (25.6)	0.008

*AKI* Acute kidney injury*, WBC* White blood cell*, ALT* Alanine aminotransferase*, AST* Aspartate transaminase*, BUN* Blood urea nitrogen, *Scr* Serum creatinine*, HDLC* High density lipoprotein*, LDLC* Low density lipoprotein, *PT* Prothrombin time, *CK* Creatine kinase

We then evaluated the effects of the independent risk factors on the occurrence of stage II-III AKI, using univariate and multivariate logistic regression. Multivariate logistic regression highlighted several distinguishing variables between the two groups, including hypertension (OR = 2.702; 95% CI 1.048–6.968), elevated WBC count (OR = 1.054; 95% CI 1.010–1.100), triglycerides (OR = 1.260; 95% CI 1.090–1.457), HDLC (OR = 0.318; 95% CI 0.139–0.726) and serum phosphorus (OR = 5.727; 95% CI 2.869–11.430), 5000 < CK ≤10,000 U/L (OR = 2.617; 95% CI 1.089–6.289) and CK>10,000 U/L (OR = 8.093; 95% CI 3.679–17.799) (Table 3). Similarly, risk factors for in-hospital mortality in patients with RM-induced AKI were analyzed. Age ≥ 60 years (OR = 2.946; 95% CI 1.072–8.097), sepsis (OR = 3.206; 95% CI 1.351–7.609) and elevated prothrombin time (OR = 1.079; 95% CI 1.004–1.160) were identified as independent risk factors, which may be associated with increased mortality rates among these patients (Table 4).

**Table 3:** Multivariate logistic regression analysis of risk factors for stage II-III AKI

Variables	B	SE	Wald	***p***	OR
Hypertension	0.994	0.483	4.229	0.040	2.702 (1.048–6.968)
WBC	0.053	0.022	5.959	0.015	1.054 (1.010–1.100)
Triglycerides	0.231	0.074	9.758	0.002	1.260 (1.090–1.457)
HDLC	−1.147	0.422	7.394	0.007	0.318 (0.139–0.726)
Phosphorus	1.745	0.353	24.499	0.000	5.727 (2.869–11.430)
5000 < CK ≤10,000	0.962	0.447	4.625	0.032	2.617 (1.089–6.289)
CK>10,000	2.091	0.402	27.033	0.000	8.093 (3.679–17.799)

*AKI* Acute kidney injury, *WBC* White blood cell*, HDLC* High density lipoprotein, *CK* Creatine kinase

**Table 4:** Risk factors for in-hospital mortality in patients with RM-induced AKI according to multivariate logistic regression analysis

Variables	B	SE	Wald	***p***	OR
Age ≥ 60	1.08	0.516	4.388	0.036	2.946 (1.072–8.097)
Sepsis	1.165	0.441	6.978	0.008	3.206 (1.351–7.609)
PT	0.076	0.037	4.289	0.038	1.079 (1.004–1.160)

*RM* Rhabdomyolysis, *AKI* Acute kidney injury*, PT* Prothrombin time

## Discussion

AKI is independently associated with the mortality rates of patients with RM. Our results have identified several independent risk factors for secondary stage II-III AKI, including hypertension, CK>5000 U/L, decreased HDLC, high levels of white blood cell count, triglycerides and serum phosphorus. Age ≥ 60 years, sepsis and elevated prothrombin time were all associated with increased mortality rates. Moreover, patients with RM resulting from sepsis, bee sting and acute alcoholism were at a higher risk of developing stage II-III AKI.

The causes of RM can be classified in a number of ways. According to the mechanisms of skeletal muscle damage, the causes have been categorized into four mechanisms: Hypoxic, physical, chemical and biological [4]. Other classification categories include surgical/medical, acquired/inherited and physical/non-physical [1, 16, 17]. In the present study, the most common cause of RM was trauma (a condition largely associated with RM [1, 2]), followed by sepsis and bee sting. Compared with other causes, patients with RM resulting from sepsis, bee sting and acute alcoholism were observed more frequently in the severe AKI group. In our study, the morbidity rate of AKI was 61.4%, which is almost in accord with previous reports [7–11], although this number varies between different studies.

Patients with RM-related AKI are associated with an increased risk of death and total health-related costs than those who do not develop AKI. Our analysis showed that the total mortality rate of these patients was 19.8%, whereas AKI patients experienced a significantly higher mortality rate than those without it (24.8% vs 11.8%, *P* < 0.01), which was within the wide range of reported mortality rates for his condition [9, 12, 13].

Different study populations (such as those with RM associated with drug use, trauma, wasp stings, infection and hospitalization) play an important role in the variations in AKI-associated morbidity, and the subsequent mortality rates of RM patients. In addition, different inclusion criteria (such as elevated CK, race and the definition of AKI itself) may also affect these results. A previous study reported that the incidence of AKI was highest according to the KDIGO definition, followed by the Acute Kidney Injury Network criteria and the Acute Dialysis Quality Initiative’s RIFLE criteria [18].

We revealed that hypertension was one of the independent risk factors for stage II-III AKI in RM patients; likewise, a previous study reported that diagnosed hypertension was an independent risk factor for AKI in patients with chronic kidney disease or after emergency department contrast-enhanced computerized tomography [19, 20]. In addition, a retrospective study of 43,611 patients demonstrated that the occurrence of AKI in hospitalized, previously normotensive adults was independently associated with increased blood pressure during the first 2 years after discharge [21]. However, there are also reports that patients with hypotension are at an increased risk of developing renal failure, decreased renal perfusion aggravate renal function [22, 23]. Long lasting hypertension damages renal blood vessels. Hypertensive patients with renin-angiotensin-aldosterone system blockade carry high risk when suffer episode of secondary AKI [24, 25]. Overexpression of reactive oxygen species caused by angiotensin 2 and decreased production of nitric oxide potently participate in the kidney ischemic injury in hypertensive surroundings [26, 27].

Low levels of HDLC have been associated with worse short-term and long-term renal outcomes in patients who suffer AKI caused by sepsis and underwent percutaneous kidney biopsy for histological diagnosis of AKI [28, 29]. In addition, HDLC was observed to have superior predictive ability than routine clinical markers for development of multiorgan dysfunction syndrome [30]. We firstly demonstrated that decreased HDLC was an independent risk factor for RM-associated stage II-III AKI. Apolipoprotein A-I mimetic peptide 4F inhibiting inflammatory responses, strengthening the vascular barrier and protecting kidney in an HDL-dependent manner was observed in animal experiment [31].

Hyperphosphatemia is a common complication of RM with several proposed pathogenic factors, including the release of inorganic phosphorus into the plasma and reduced urinary phosphate excretion [32, 33]. In the present study, increased serum phosphate was determined to be an independent predictor for AKI-associated RM, which is in accordance with previously reported findings [11, 34, 35]. Furthermore, phosphate has also been verified as a potential biomarker of disease severity and prognosis in AKI patients undergoing continuous renal replacement therapy [36]; this phenomenon was not observed in our study, which included patients with AKI of all stages.

The significant correlation between serum CK level and the risk of RM-induced AKI has been demonstrated in previous studies [37–39], our analysis confirmed that. Moreover, different classifications (5000 < CK ≤10,000 U/L, CK>10,000 U/L) have different risks for stage II-III AKI in patients with RM. While, elevated serum CK is not associated with the mortality rate of patients with AKI.

### Study limitations

The present study includes the following limitations. Firstly, most of the recruited patients were identified by discharge diagnoses in an electronic medical record database; hence, a proportion of the patients with elevated serum CK were missed due to a lack of RM diagnosis. Secondly, where existing urine volume data were not available, AKI diagnosis was based on SCr alone; thus, the incidence of AKI may have been underestimated. Thirdly, some of the possible risk factors for AKI, such as the level of myoglobin, lactic acid, associated drugs, as well as the severity-of-illness scores, were not included due to incomplete data. Finally, no follow-up study was conducted; thus, long-term patient prognoses (including recovery of renal function, risk of recurrent AKI and late mortality) were not determined.

## Conclusion

The occurrence of AKI increases the mortality rate of patients with RM. The early evaluation and diagnosis are crucial for the prevention of AKI and improved patient prognosis. Our study demonstrated several risk factors for RM-induced stage II-III AKI and in-hospital mortality in RM patients with AKI. These findings may facilitate the effective prevention and management of RM patients with AKI. However, it also has several limitations. We will conduct further research to improve the study.

## Data Availability

The datasets generated and/or analysed during the current study are not publicly available due to privacy but are available from the corresponding author on reasonable request.

## Abbreviations

RMf: Rhabdomyolysis
CK: Creatine kinase
AKI: Acute kidney injury
SCr: Serum creatinine
BUN: Blood urea nitrogen
WBC: White blood cell
KDIGO: Kidney Disease Improving Global Outcomes
SD: Standard deviation
OR: Odds ratio
ALT: Alanine aminotransferase
AST: Aspartate aminotransferase
HDLC: High-density lipoprotein cholesterol
LDLC: Low-density lipoprotein cholesterol
PT: Prothrombin time
